# Application of Essential Oils Extracted from Peel Wastes of Four Orange Varieties to Control Anthracnose Caused by *Colletotrichum scovillei* and *Colletotrichum gloeosporioides* on Mangoes

**DOI:** 10.3390/plants12152761

**Published:** 2023-07-25

**Authors:** Chau Trung Duong, Huynh Thi Phuong Thao, Nguyen Thi Nhu Y, Doan Thi Kieu Tien, Nguyen Thi Thu Nga, Tran Chi Nhan, Bui Thi Cam Huong, Sezai Ercisli, Nguyen Thi Ngoc Truc, Luu Thai Danh

**Affiliations:** 1 Can Tho Technical Economic College, Can Tho 94000, Vietnam; ctduong@ctec.edu.vn; 2 Faculty of Applied Biological Sciences, Vinh Long University of Technology Education, Vinh Long 85110, Vietnam; thaohtp@vlute.edu.vn; 3 College of Agriculutre, Can Tho University, Can Tho 94000, Vietnam; ynhunguyen1208@gmail.com (N.T.N.Y.); dtktien@ctu.edu.vn (D.T.K.T.); nttnga@ctu.edu.vn (N.T.T.N.); btchuong@ctu.edu.vn (B.T.C.H.); 4 Institute of Food and Biotechnology, Can Tho University, Can Tho 94000, Vietnam; tcnhan@ctu.edu.vn; 5 Department of Horticulture, Faculty of Agriculture, Atatürk University, Erzurum 25240, Türkiye; sercisli@gmail.com; 6 HGF Agro, ATA Teknokent, Erzurum 25240, Türkiye; 7 Southern Horticultural Research Institute, Tien Giang 84000, Vietnam; truc971976@gmail.com

**Keywords:** anthracnose, antifungal, *Colletotrichum gloeosporioides*, *Colletotrichum scovillei*, essential oil, orange peels

## Abstract

A huge amount of orange peel waste is annually discharged into the environment. Processing of this waste for the control of post-harvest fruit diseases can reduce environmental pollution. Essential oils (EOs) from fruit peels of *Citrus reticulata* × *sinensis* (Sanh cultivar) and *Citrus sinensis* (Xoan, Mat and Navel cultivar) were investigated for their ability to control anthracnose caused by *Colletotrichum gloeosporioides* and *Colletotrichum scovillei* on mangoes. EOs were extracted by hydro-distillation and analyzed by GC-MS and GC-FID. The antifungal activity of the EOs was determined by in vitro and in vivo assays. The Mat cultivar had the highest extraction yield of 3% FW, followed by Xoan (2.9%), Sanh (2.2%), and Navel (1%). The chemical composition of the EOs was similar, with limonene as the main compound (around 96%). The antifungal activity of EOs was not different, with a minimum fungicidal concentration of 16% for both fungi. The disease inhibition of EOs increased with their concentration. The highest inhibition of anthracnose caused by both fungi on mangoes was achieved at 16% EO. EOs had no adverse effect on mango quality (pH, total soluble solids, total acidity, color and brightness of mangoes), except firmness and weight loss at high concentrations (16%). Orange EOs can be used as bio-fungicides to control mango anthracnose at high concentrations.

## 1. Introduction

**Figure 1:**
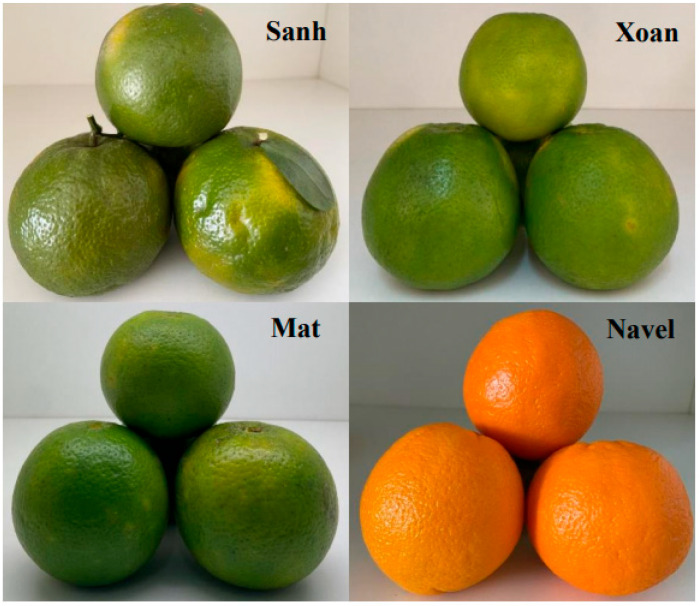
Fruits of *Citrus reticulata* × *sinensis* (Sanh variety) and *Citrus sinensis* (Xoan, Mat, and Navel varieties).

Orange is one of the most popular citrus fruits, with a total world production of about 48 million tons per year in 2022–2023, in which orange production in Vietnam was estimated to be about 1.15 million tons [1]. The Navel variety yellow orange (*Citrus sinensis*) is the most common and most widely recognized type in the world. This variety is extensively grown in sub-tropical climates, such as the Mediterranean, Spain, Morocco, Turkey, South Africa, Australia, California, Florida, Uruguay, Brazil, and Argentina [2]. In the Mekong Delta, Vietnam’s vital agricultural zone (tropical climate), there are three green orange varieties which are commonly cultivated. The Sanh variety (*Citrus reticulata* × *sinensis*) is the most popular, followed by the Xoan (*Citrus sinensis*) and Mat (*Citrus sinensis*) varieties (Figure 1).

Orange is consumed as fresh produce or can be used as a raw material for producing juice and concentrates. Fresh fruit consumption and fruit juice processing generate huge amounts of waste (about 50–60% of the fresh orange), which mostly consists of orange peels [3]. The majority of orange waste is disposed in the environment [4]; only a small amount is utilized to produce molasses, pectin, and essential oils [3]. Promoting the use of orange EOs as agrochemicals to control post-harvest fruit diseases can valorize orange waste and reduce its disposal in the environment. To date, there has been no comparative investigation into the antifungal activity of EOs from the fruit peels of green and yellow oranges against *Colletrotrichum* spp. causing mango anthracnose.

Anthracnose induces serious damage to mango fruits, especially during the post-harvest stage. According to Ann et al. [5], anthracnose on mango fruits can result in economic losses of up to 60%. Mango anthracnose is mainly caused by *Colleototrichum* spp., of which *Colleotrichum gloeosporioides* is the most common pathogen [6]. Recently, several studies have demonstrated that *Colletotrichum scovillei* is a new anthracnose-causing agent in mango fruits in China [7], Taiwan [8], and Vietnam [9].

To produce anthracnose-free mangoes, synthetic fungicides are commonly used. The application of these substances raises public concern about potentially harmful effects on human health and the environment. Therefore, research into and development of new anthracnose control products that are safe for human health and environmentally friendly have attracted great attention from the agrochemical industry and in academia worldwide. Natural plant extracts, particularly essential oils (EOs), have been demonstrated to be ideal candidates for use as agrochemicals among various alternatives, as they are highly effective, biodegradable, and eco-friendly [10]. EOs from some plant species have shown the antifungal activities against *C. gloesporioides*, which causes anthracnose on mango fruits [11,12,13]. Several studies have reported that orange EOs possess a strong toxicity capacity against *C. gloeosporiodes* in vitro, and that they are able to greatly reduce anthracnose incidence on mango fruits [10,13]. However, the orange EOs in these studies were not chemically analyzed using GC-MS or GC. In addition, the effect of orange EOs on mango quality was also not investigated. The lack of such scientific information may hinder the commercial application of orange EOs to control mango anthracnose.

This study was aimed to comparatively investigate the EO extraction yields from the fruit peels of three green (Sanh, Xoan, and Mat) and one yellow (Navel) orange by hydrodistillation; to chemically analyze orange EOs by means of GM-MS and GC; and to evaluate their ability to act against *C. gloeosporioides* and *C. scovillei* (a new mango anthracnose-causing agent) in vitro. In addition, the in vivo effects of orange EOs on the control of anthracnose development in artificially infected mangoes and the quality of the mangoes were also investigated.

## 2. Results and Discussion

### 2.1. Oil Extraction Yield 

**Figure 2:**
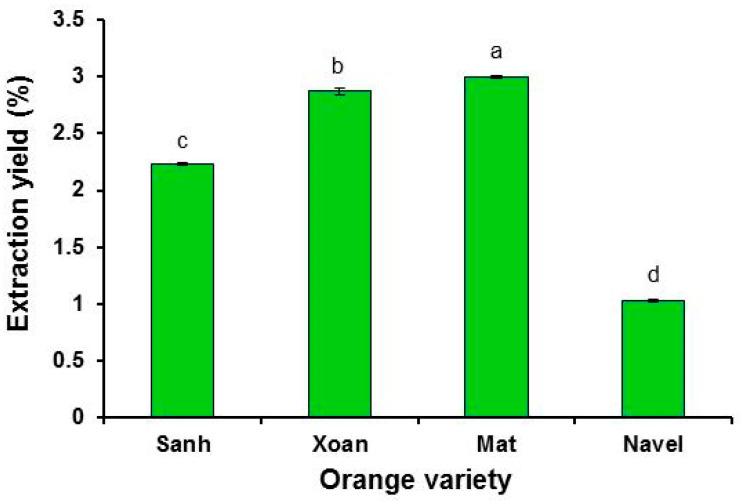
Extraction yield of four orange varieties. Different letters indicate significant differences at a 5% level of significance.

The yield of EOs extracted by hydro-distillation from fruit peels of four orange varieties is presented in Figure 2. Cold pressing is the most common method used for the commercial extraction of orange oils. However, the orange oil extraction yield from cold pressing was more than four times lower than that of hydro-distillation [14]. Therefore, hydro-distillation was selected for this study. The calculation of the extraction yield was based on the fresh weight of fruit peels. The yields were significantly different among the four varieties; Mat oranges had the highest yield (3%), followed by Xoan (2.87%), Sanh (2.23%), and Navel (1%). The green and yellow varieties had oil yields within the oil yield range (0.46–4%) recorded in the literature [15,16,17]. The variation in the orange oil yields among the scientific reports was due to the differences in genetics (species or varieties), growth environment, cultivation practice, storage time, sample preparation, and extraction method. 

The oil yields of Mat, Xoan, and Sanh (green varieties) were more than two times higher than those of Navel oranges (yellow variety). Navel oranges, in this study, had smaller oil yields than the Navel oranges (1.7%) reported in the study of Bustamante et al. [16]. The low extraction yield of Navel oranges in this study may be due to the long storage time. Navel oranges are commonly imported from Australia to Vietnam by sea, and the shipping time is about 22–30 days. Upon arriving in Vietnam, the oranges were transported to a supermarket and kept there for a while before purchased for this study. The estimated time from picking up the Navel oranges at orange farms in Australia to the oranges arriving at the laboratory was more than 30 days. For the green oranges (Xoan, Sanh, and Mat), this took only a few days. The longer the storage time for the oranges, the lower the content of EOs in fruit peels. Orange oils are one of the most volatile oils. Under an open environment and high temperature (above 0 °C), orange oils can be quickly evaporated into the air. This resulted in the low extraction yield of Navel oranges in this study. Furthermore, under visual observation, the layers of albedo (the inner white part) in the fruit peels of Sanh and Navel oranges were thicker than those of Mat and Xoan oranges. On the contrary, the flavedo (the outer colored part containing the oil sacs) layers of Mat and Xoan fruit peels were thicker than those of Sanh and Navel oranges. These characteristics may contribute to the lower oil yield of Sanh and Navel than that of Mat and Xoan. 

### 2.2. Chemical Analysis of Orange Essential Oils 

The chemical components and their relative concentrations (%) of orange oils are shown in Table 1. In general, the chemical compositions of orange oils from green (Sanh, Xoan, and Mat) and yellow (Navel) varieties were similar. Limonene was the most abundant chemical compound in all orange oils, with a concentration of about 96% of the oil content. Its relative concentration was statistically similar among all of the tested oils. Other minor components detected in the four orange oils included *β*-myrcene (1.87–1.99%), *α*-pinene (0.51–0.58%), linalool (0.02–0.39%), *β*-pinene (0.13–0.28%), and *β*-carene (0.14–0.27%). The main compounds of orange oils in this study were also detected in the previous studies [16,18,19,20], and had concentrations of limonene (91.1–98.4%), *β*-myrcene (0.03–1.98%), *α*-pinene (0.22–0.53%), linalool (0.04–2.56%), *β*-pinene (0.03–1.82%) and *β*-carene (0.01–0.22%) within the reported ranges. The reported chemical composition of orange oils varied between different scientific reports, owing to discrepancies in genotypes, growth conditions, farming practices, and plant sample preparation and extraction methods. 

In this study, limonene may possibly have been responsible for the biological activities and applications of orange oils, as it accounted for nearly 100% of the content of the orange oils (Table 1). Limonene in orange EO was demonstrated to be responsible for the antifungal activity against *C. gloeosporioides*, which caused mango anthracnose in the study by Combrinck et al. [11]. Orange oils with 95% limonene had the same MIC of 3 µL/mL as limonene (98% purity). Limonene is widely used as a flavor and fragrance additive in foods, beverages, perfumes, soaps, detergents, and household cleaning products due to its pleasant citrus fragrance [21]. Limonene has been demonstrated to possess a wide range of biological activities, namely, antibacterial, antifungal, antifeedant, antioxidant, anti-inflammatory, anticancer, and antinociceptive activities [21]. In addition, orange oil (main source of limonene) is one of the most abundant oils with a low price. These conditions extend the application possibilities of orange oils in the medicine, cosmetic, and agrochemical industries. 

### 2.3. In Vitro Antifungal Activity of Orange Oils against C. gloeosporioides and C. scovillei

The in vitro antifungal activity of orange oils, expressed by the inhibition zone, is presented in Table 2. The larger the diameter of the inhibition zone, the stronger the antifungal capacity. DMSO (an antimicrobial agent), when used to dissolve orange oils in testing solutions, had no inhibitory effect on the mycelial growth of *C. gloeosporioides* or *C. scovillei*. Therefore, orange oils were solely responsible for the antifungal activity in this study. The fungal toxicity of orange oils was dose-dependent. Both fungi showed inhibition zones that increased with the concentration of orange oils. *C. gloeosporioides* was more sensitive to oils of Sanh, Xoan, and Mat oranges than *C. scovillei* at high concentrations. The diameter of the inhibition zone induced by these oils at the concentration of 8 µL/paper disc on *C. gloeosporioides* was in range of 7.9–10 mm greater than that of *C. scovillei* (1.6–3.7 mm). In addition, at this concentration, the inhibition zone of *C. gloeosporioides* decreased in the order of Sanh, Xoan, Mat, and Navel orange, while the opposite trend was observed for *C. scovillei*.

The MIC of all tested oils against *C. gloeosporioides* was the same as that of *C. scovillei* (Table 3). For both fungal species, the MICs of Sanh and Navel (8% *v*/*v*) were two times higher than those of Xoan and Mat (4% *v*/*v*). The chemical composition of EOs determines their biological activities. The fungistatic capacity of Sanh and Navel was weaker than that of Xoan and Mat. It is possible that minor components undetected by chromatography analysis in Xoan and Mat oils may have been responsible for the higher fungistatic (temporarily inhibitory) capacity observed in these oils.

Four orange oils had MICs against *C. gloeosporioides* which were higher than reported in previous studies. The MIC of orange (*Citrus sinensis*) oil according to the studies of Combrinck et al. [11] and Abd-Alla and Haggag [10] were 3 µL/mL (equivalent to 0.3% *v*/*v*) and 150 µg/mL (equivalent to 0.018% *v*/*v*), respectively. The large difference between the MIC of this study and those of the previous studies can be explained by two possible reasons. Firstly, the orange oil in the previous reports was incorporated into solid fungal growth medium (PDA), while in this study, the oils were mixed into a liquid medium (PDB). Orange oils (mainly limonene) have very high volatility. The solid matrix of PDA would hinder the evaporation of orange oils into the air; thus, the concentration of orange oil in the testing medium may show a small reduction over time. On the contrary, orange oils in the liquid environment may quickly vaporize into the air, resulting in large decreases in their content. Consequently, the assay using the liquid medium may require higher orange oil concentrations than that of the solid medium to completely inhibit fungal growth. Secondly, *C. gloeosporioides*, when exposed to an environment containing vapors of orange oil (limonene) for a long time, may gradually develop resistance to this oil. *C. gloeosporioides* isolated from anthracnose mangoes was collected in the Mekong Delta. In this region, farmers commonly own small areas of land (less than 1 hectare). Mango farms and citrus (orange, pomelo, lemon, and mandarin) farms are often located side by side. Some farmers cultivate a wide range of fruit trees in their farms, including mango, orange, pomelo, mandarin, and lemon. Therefore, there is a great chance for *C. gloeosporioides*, a pathogen of mango anthracnose, to come into close contact with *Citrus* plants. Limonene is the main component of essential oils in the peels of not only oranges, but also other citrus fruits. Limonene is also present in high concentrations in the EOs of flowers and leaves of *Citrus* spp. [21]. The compound emitted from plant parts of *Citrus* spp. has created great pressure on *C. gloeosporioides*, which is present in mango plants nearby, to develop resistance. The chance of isolating *C. gloeosporioides* which was less sensitive to orange oils was quite high in this study. Therefore, MIC against *C. gloeosporioides* in this study was much higher than that in Combrinck et al. [11] and Abd-Alla and Haggag [10]. A similar phenomenon may occur with *C. scovillei* that builds up its resistance to orange oil (limonene) over a long exposure period. 

The MFC values of the four orange oils were the same (16%) for *Colletotrichum* spp. (Table 3), because the chemical composition of the orange oils was similar, and their limonene contents (about 96%) were insignificantly different (Table 1). In the study of Combrinck et al. [11], limonene and orange oil (95% limonene) had the same MFC (3 µL/mL) against *C. gloeosporiodes* isolated from anthracnose mangoes. These results indicate that limonene is responsible for the fungicidal (fungi-killing) activity of orange oils. The MFC values were higher in this study than in the report by Combrinck et al. [11], which can be clarified by the fact that *C. gloeosporioides* (and *C. scovillei*) may have been less vulnerable to orange oil toxicity in this study owing to the development of orange oil resistance, as discussed above. 

### 2.4. In Vivo Assay of Orange Oils against Anthracnose Development in Mangoes

The data presented in Table 4 show disease inhibition (%) of orange oils against anthracnose caused by two fungi. The MIC of orange oils was selected for the in vivo assay. In addition, the MFC was the same for all of the orange oils tested against the fungi, and Sanh oranges were the most abundant variety in Mekong Delta. Therefore, Sanh oil with a concentration of 16% *v*/*v* (MFC) was chosen for the study. The appearance of anthracnose was monitored daily, and disease lesions appeared at 4 days after inoculation (DAI). 

**Figure 3:**
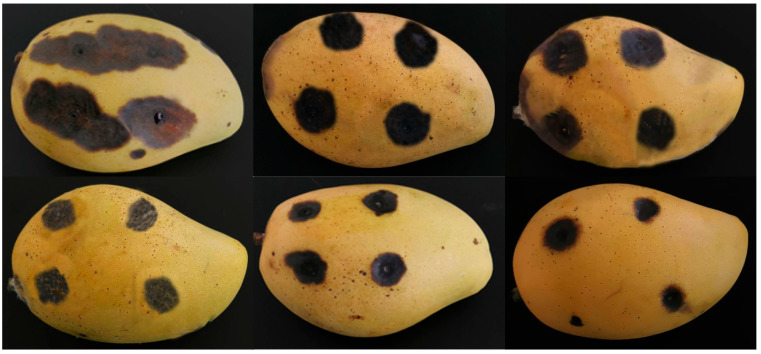
Anthracnose lesions on mangoes artificially infected by *C. gloeosporioides* under control and orange oil treatments at 8 days after inoculation. Top row, from left to right: distilled water, 4% Mat, and 4% Xoan oil treatments. Bottom row, from left to right: 8% Navel, 8% Sanh, and 16% Sanh oil treatments.

Different orange oils at the same concentrations (Xoan and Mat at 4%; Navel and Sanh at 8%) generally had similar percentages of disease inhibition over 8 DAI (Table 4 and Figure 3). The chemical compositions of the orange oils were indistinguishable, with limonene (the main fungicidal agent) accounting for about 96% of the oil content of the four orange oils. Thus, they may have similar effects in terms of controlling anthracnose development. Disease inhibition increased with the orange oil concentration regardless of the orange oil type. Sanh oil, at a concentration of 16%, had the highest percentage of disease inhibition—in the range of 39.5–49.4% for *C. gloeosporioides* and 30.4–47.5% for *C. scovillei*. In the study conducted by Abd-Alla and Haggag [10], orange oil at a concentration of 1 µL/mL (equivalent to 0.1%) showed disease inhibition of 91.3% against anthracnose caused by *C. gloeosporioides* in mangoes. The lower disease inhibition at higher oil concentrations found in this study, as compared to the Abd-Alla and Haggag [10] study, may have been due to the resistance of anthracnose causing pathogens towards orange oils (limonene), as discussed above, and differences in the storage temperature. The study by Abd-Alla and Haggag [10] was conducted at 13 ± 1 °C, while the temperature in this study was kept at 28 ± 2 °C. At low temperatures, *C. gloeosporiodes* was less active, and the orange oils showed low volatilization. Such conditions made orange oil treatment in the previous study more effective than in this study. 

In this study, orange oils showed moderate fungistatic and fungicidal activity against the growth of *C. gloeosporioides* and *C. scovillei* (Table 2 and Table 3). Consequently, they had a moderate effect on controlling the development of anthracnose caused by *C. gloeosporioides* and *C. scovillei* on mangoes (Table 4). To increase the effectiveness of anthracnose control, orange oils can be mixed with other EOs to exploit the interactive effect of the EO mixture. They can also be incorporated into polymers, such as chitosan, to reduce volatility. After the treatment of orange oils, it is suggested that mangoes should be stored at cool temperatures to hinder anthracnose development. 

### 2.5. Effect of Orange Oils on Mango Quality

The quality parameters of Cat Chu mangoes under treatments with Sanh EO are presented in Table 5. Only Sanh EO was used in this study, because Sanh oranges are the most common variety cultivated in the Mekong Delta, and the chemical compositions of the four orange EOs were similar. A 5% DMSO solution (used to dissolve orange oils in the testing solutions) and distilled water were assigned as the control treatments. 

**Figure 4:**
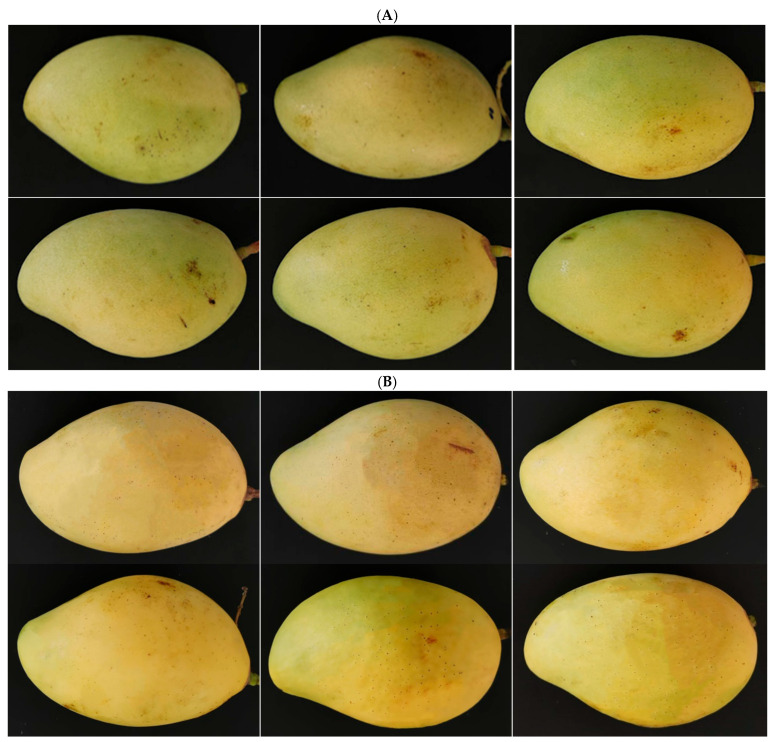
Mangoes’ appearance before and after 8 days of treatment. (**A**) Mangoes before treatment. (**B**) Mangoes after 8 days of treatment. Top row, from left to right: 5% DMSO, distilled water, and 2% oil treatment; bottom row, from left to right: 4%, 8%, and 16% oil treatment.

Sanh orange oil, even at a high concentration of 16%, had a negligible effect on mango fruit quality. The pH, total soluble solids (TSS), and titratable acidity (TA) of the fruit pulps were statistically indistinguishable between all oil and control treatments after 8 days of storage. There was no significant difference among oil treatments in terms of fruit firmness. However, the fruit firmness achieved by the oil treatments (2.42–2.56 kgf/cm^2^) was slightly harder than that with the control treatments (2.07–2.15 kgf/cm^2^). The results were consistent with the findings of Sefu et al. [22] that the firmness of mangoes treated with cinnamon and ginger EOs was higher than that of control treatments after 10 days of storage. EOs may interfere with the activity of enzymes responsible for the degradation of fruit cell walls during the ripening process. The fruit peel colors were evaluated using the Commission Internationale de l’Eclairage (CIE) system: L* (0: dark; 100: white), a* (negative value: green; positive value: red) and b* (negative value: blue; positive value: yellow). The oil treatments had similar L* and a* values as those of the control treatments; however, their a* values were marginally lower than those of the control treatments. The results indicated that the fruit peel colors were generally the same with the oil and control treatments, but the control fruit peels were more red than the oil-treated ones. The appearance of mangoes under orange oil treatments at various concentrations was generally similar to that of the control-treated mangoes (Figure 4). In short, orange oils, at various concentrations, did not induce any significant changes in the pH, TSS, or TA of fruit pulps, nor in the fruit peel color or appearance. 

The effect of Sanh orange oil at different concentrations on weight loss in mangoes is shown in Table 6. Weight loss increased in mangoes under all treatments with storage times over 8 days. The findings are in agreement with the study of Van et al. [23]. Weight loss with treatments between 2% and 4% was insignificant at four sampling points. A similar result was also observed for 8% and 16% treatments. The weight loss of mangoes treated with 8 and 16% was slightly higher than that of control treatments. In contrast, the weight loss with 2 and 4% treatments was marginally lower than that of the control treatments. Treatments at low concentrations (2 and 4%) reduced weight loss, while high-concentration treatments (8 and 16%) increased weight loss. The results of this study show that orange oil had only a slight effect on weight loss in mangoes. 

## 3. Materials and Methods

### 3.1. Sample Collection

Ripe oranges of three green varieties, namely, Sanh (*Citrus reticulata* × *sinensis*), Xoan (*Citrus sinensis*), and Mat (*Citrus sinensis*), were supplied by growers in the Mekong Delta. Navel oranges (*Citrus sinensis*) imported from Australia were purchased from the local supermarket in Can Tho City, Vietnam. The oranges were washed using tap water, then air-dried. Peels (flavedo and albedo) were removed and kept in air-tight plastic bags, which were stored at −20 °C until they were used for EO extraction. The moisture content of the fresh peels was determined by drying in the oven at 70 °C until the peel weights were unchanged. Fresh peels of Sanh, Xoan, Mat, and Navel oranges had moisture contents of 78.9, 69.1, 74.5, and 72.2%, respectively. Moreover, physiologically mature mangoes of the Cat Chu variety were bought from a grower in the Mekong Delta, Vietnam.

### 3.2. Essential Oil Extraction

Two hundred grams of orange peels were cut into small pieces of about 2 cm × 2 cm. Subsequently, plant materials were blended with 500 mL of deionized water. Mixtures of samples and water were hydro-distilled using a Clevenger apparatus for 3 h. The oils were collected and water was removed by anhydrous sodium sulfate. The EOs were weighed and stored in air-tight amber glass vials at −20 °C until further use. Extraction was repeated three times for each orange variety. The extraction yield was calculated using the following equation:

**Extraction yield** (**%**) = 100% × weight of EO (g) / weight of orange peels (g)

### 3.3. Chemical Analysis of Essential Oils

The components of orange EOs were chemically identified by comparing their mass spectral and Kovats retention indices (KI) with those recorded in the Adam library of EO components [24] and the NIST mass spectral library. Mass spectra of the EO components were determined by gas chromatography–mass spectrometry (GC-MS) analyses on a Thermo Scientific (Waltham, MA, USA) instrument equipped with a HP-5MS (5% phenyl, 95% dimethylpolysiloxane) capillary column (30 m × 0.25 mm i.d. × 0.25 m film thickness). Sample volumes of 1 µL were injected using the split mode (split ratio 1:10). Helium was used as a carrier gas with a flux of 1.1 mL/min. The injector and detector temperatures were set at 230 and 275 °C, respectively. The thermal program of the oven was initially set at 50 °C for 5 min, increased to 180 °C (at the rate of 3 °C/min) then to 250 °C (at the rate of 10 °C/min), and was held for a further 5 min. KI was determined for the individual components by using the retention times of a series of n-alkanes (C7–C30) under the same instrument and analysis conditions. 

The relative concentration (%) of orange oil components was determined by a gas chromatography–flame ionization detector (GC-FID) using a Shimadzu GC 2030 equipped with an AT-5 (5% phenyl, 95% dimethylsiloxane) capillary column (30 m × 0.25 mm i.d. × 0.25 m film thickness). Nitrogen was used as the carrier gas with a flow rate of 1 mL/min. Samples of 1 µL were injected using the split mode (split ratio 1:10). The thermal program was similar to the GC-MS analysis above. The percentage of oil components was calculated from electronic integration measurements based on area normalization.

### 3.4. Pathogen Isolates

**Figure 5:**
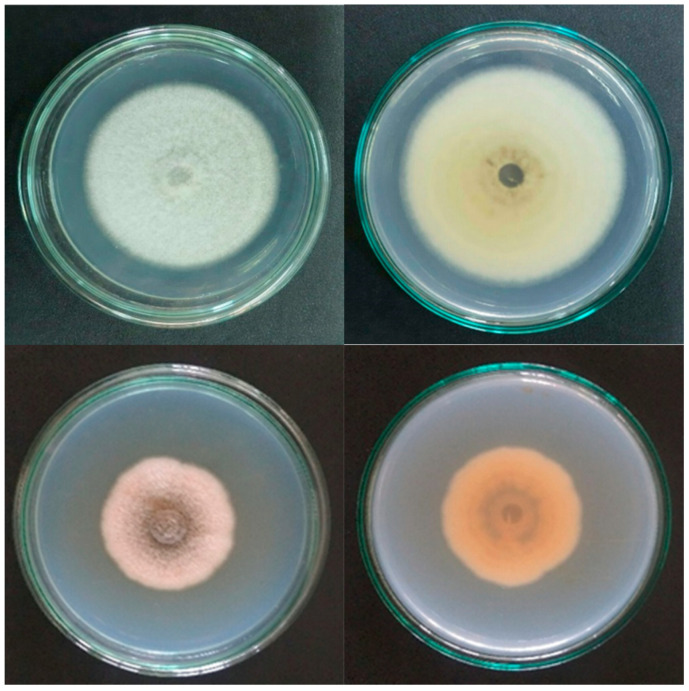
Mycelial growth of *C. gloeosporioides* (top row) and *C. scovillei* (bottom row) on 90 mm petri dishes at 7 days after culture. Upper and lower colony surfaces are in the right and left columns, respectively.

*C. gloeosporioides* and *C. scovillei* were isolated from symptomatic mangoes of the Cat Chu variety, then identified by morphology and DNA sequencing of the ITS region in our previous study [9]. Fungi were cultured on potato dextrose agar (PDA) at 30 °C to activate spore formation (Figure 5). The surfaces of the agar plates were washed with 10–20 mL sterile distilled water to collect spore suspensions, which were then passed through two layers of sterile cloth. The volume of suspension was adjusted with sterile distilled water in order to achieve a spore density of 10^6^ cfu/mL, which was confirmed using a hemocytometer.

### 3.5. In Vitro Assays of Antifungal Activity

The antifungal activity of orange oils against *C. gloeosporioides* and *C. scovillei* was determined by the agar plate diffusion method [25]. The oils were diluted in a solution of 5% dimethyl sulfoxide (DMSO) to obtain concentrations of 20, 40, and 80% *v*/*v*. The surface of the agar plates was evenly spread with 50 µL of spore suspension (10^6^ cfu/mL), then air-dried. Next, 10 µL of oil or a control solution was added to a sterile filter paper disc 6 mm in diameter, which had previously been placed on the agar plate containing fungi. Fungicide (Antracol 70 WP with 70% active ingredient of probineb) at a concentration of 6 mg/mL and pure DMSO were used as positive and negative controls, respectively. Plate incubation was performed at 30 °C for 30 min, then at 37 °C for 48 h. After incubation, the zone of inhibition was measured in millimeters. The experiment was repeated 5 times for each EO. 

The minimum inhibitory concentration (MIC) of orange EOs was determined using the microdilution method [25], and 75 µL of spore suspension (10^6^ cfu/mL) was added to wells of a 96-well plate containing 75 µL of potato dextrose broth (PDB) with different concentrations of orange oils (0.5, 1, 2, 4, 8, and 16% *v*/*v*) and 5% DMSO. The control treatments included: (1) negative control (only 150 µL PDB); (2) positive control 1 (75 µL spore suspension and 75 µL PDB); and (3) positive control 2 (75 µL spore suspension and 75 µL PDB containing 5% DMSO). The experiment was performed with 5 replicates for each oil concentration. The plate was incubated at room temperature. At 0 and 24 h after incubation, the growth of the fungi was visibly recorded and measured at a wavelength of 492 nm using a spectrometer (Thermo Scientific, Waltham, MA, USA). The lowest concentrations of orange essential oils that completely inhibited fungal growth were defined as the minimum inhibitory concentrations (MIC). 

The minimum fungicidal concentration (MFC) was determined by re-inoculating the fungal solutions from the MIC assay above on PDA plates. The MFC value is the lowest concentration of essential oil showing no growth or less than three fungal colonies (killing activity: about 99% to 99.5%) after 96 h of re-inoculation on the agar plate [26].

### 3.6. In Vivo Assay of Inhibiting Anthracnose Development on Mango Fruits

The inhibitory capacity of orange oils against anthracnose caused by *C. gloeosporioides* and *C. scovillei* on Cat Chu mangoes was investigated by the method described in the study of Danh et al. [25]. The physiologically mature and healthy mangoes with uniform sizes and colors were selected for the study. The fruits were initially washed under running tap water, then disinfected with 70% ethanol and left to dry naturally. The fruits were subsequently wounded by piercing a bunch of needles at 4 sites to create wounds 2 mm deep. Orange oil solutions containing 5% DMSO, 15 µL in volume, were applied to the wounds and allowed to dry for 1 h. Thereafter, a spore suspension (10^6^ cfu/mL) at the volume of 15 µL was applied on the treated wounds. Distilled water, DMSO, and Antracol 70 WP (6 mg/mL) were used as control treatments. Mangoes were kept at 28 ± 2 °C for 8 days. Ten replicates were used for each treatment with one fruit per replicate. Anthracnose lesion diameters were recorded at 4, 6, and 8 days after treatment.

**Disease inhibition** (**%**) = [Lesion diameter of control (water) treatment − lesion diameter of EO treatment] × 100% / [Lesion diameter of control (water) treatment]

### 3.7. Effect of Orange Essential Oils on the Fruit Quality of Cat Chu Mangoes

Healthy and mature mangoes were immersed in solutions containing 5% DMSO and orange oil at various concentrations for 30 s, then stored at 28 ± 2 °C for 8 days. Distilled water and 5% DMSO solution were used as control treatments. There were 10 replicates for each essential oil concentration and control treatment, with one fruit per replicate. The quality parameters of mangoes, namely, the color, fruit firmness, total dissolved solids (TSS), pH, and titratable acidity (TA), were measured at 8 days after storage. The peel fruit color was measured using a colorimeter (Konica Minolta CR-20, Osaka, Japan). Measurements were performed at the head, middle, and tail positions of the mangoes, and expressed as mean values. Measurements of fruit firmness were performed using a fruit sclerometer (Lutron FR-5105, Taiwan). The hardness was based on the force (kgf/cm^2^) required for a 6 mm penetrometer tip to puncture mango fruits at depths of 5 mm at 3 different sites, and was shown as an average value. The fruit pulp was pureed in a blender without adding water, then its pH and total dissolved solids (TSS) were determined using a pH meter and a refractometer, respectively. The titratable acidity of the fruit pulp was determined by titrating 10 mL of fruit juice with 0.1 N sodium hydroxide in the presence of phenolphthalein to indicate the endpoint [27]. The weights of the mangoes were determined at 48 h intervals over 8 days of storage, and weight loss percentages were calculated using these data.

### 3.8. Statistical Analysis

Data were input into Excel 2010 sheets and subsequently processed using IBM SPSS Statistics for Windows version 21 (IBM Corp., Armonk, NY, USA) for one-way analysis of variance. The treatment means were ranked using Duncan’s test at a 5% level of significance.

## 4. Conclusions

The study extracted essential oils from orange fruit peels of three green (Sanh, Xoan, and Mat) and one yellow (Navel) variety. The oil yield (fresh weight basis) of Mat was the highest, followed by that of Xoan, Sanh, and Navel. The oils of the green and yellow orange varieties had the similar chemical compositions, in which limonene was the main component, accounting for about 96% of the oil content. The fungistatic (inhibitory) activity, expressed by inhibition zone and MIC, against *C. gloeosporioides* and *C. scovillei* varied among the orange oils. However, the fungicidal (killing) activity of four orange oils, demonstrated by MFC, was the same. The inhibition of anthracnose development in artificially infected mangoes increased with the concentration of orange oils. The highest disease inhibition was obtained at 16% orange oil treatment, with 39.5–49.4% for *C. gloeosporioides* and 30.4–47.5% for *C. scovillei*. Orange oils had a moderate control effect on anthracnose development in Cat Chu mangoes. At various concentrations, the orange oil had a negligibly negative effect on the pH, TSS, and TA of fruit pulps; the fruit peel color; the fruit firmness; and weight loss. In short, according to the results obtained in this study together with their great abundance and low prices, orange oils can be commercially used at high concentrations (up to 16%) to protect Cat Chu mangoes from anthracnose caused by *C. gloeosporioides* and *C. scovillei.* To increase the effectiveness of anthracnose control, orange oils can be formulated with polymers, such as chitosan, to reduce their volatility, or mixed with other EOs to exploit the interactive effects of essential oil combinations.

## Figures and Tables

**Table 1:** Chemical composition of orange essential oils.

No.	KI	Compound	Relative Concentration (%)				Statistic Analysis
			Sanh	Xoan	Mat	Navel	
1	932	*α*-pinene	0.58 ± 0.02	0.58 ± 0.00	0.57 ± 0.00	0.51 ± 0.00	Ns
2	968	Sabinene	0.13 ± 0.01	-	-	-	
3	972	*β*-pinene	0.13 ± 0.01	0.19 ± 0.00	0.18 ± 0.00	0.28 ± 0.00	*
4	994	*β*-myrcene	1.94 ± 0.09	1.95 ± 0.00	1.99 ± 0.01	1.87 ± 0.01	Ns
5	1005	δ-3-carene	0.14 ± 0.00	0.14 ± 0.00	0.27 ± 0.00	0.15 ± 0.00	*
6	1031	Limonene	96.2 ± 0.19	96.5 ± 0.02	95.9 ± 0.27	95.5 ± 0.17	Ns
7	1058	*γ*-terpinen	-	0.01 ± 0.00	-	0.01 ± 0.00	
8	1089	Terpinolene	-	-	0.04 ± 0.00	0.30 ± 0.01	
9	1104	Linalool	0.20 ± 0.00	0.02 ± 0.00	0.39 ± 0.00	0.27 ± 0.01	*
10	1181	Verbenol	0.01 ± 0.00	0.01 ± 0.00	-	0.12 ± 0.00	
11	1193	*cis*-piperitol	0.03 ± 0.01	-	-	0.01 ± 0.00	
12	1194	*α*–terpineol	-	-	0.03 ± 0.00	-	
13	1378	Geranyl acetate	-	-	-	0.13 ± 0.00	
**Total % identified**			99.36	99.40	99.37	99.15	

Note: Data are shown as the mean values of three replicates ± SD. In the same row, Ns stands for not significantly different, and * stands for significantly different at 5%. -: undetected.

**Table 2:** Diameter of inhibition zones induced by orange oils (mm).

Treatment	*C. gloeosoprioides*			*C. scovillei*		
	Essential Oil Concentration (µL/Paper Disc)					
	2	4	8	2	4	8
Sanh	0	3.2 ± 0.5	10 ± 0.2	0	0.8 ± 0.1	1.6 ± 0.1
Xoan	0	1.5 ± 0.3	8.6 ± 0.7	0.8 ±0.1	1.5 ± 0.1	2.4 ± 0.1
Mat	0	1.9 ± 0.3	7.9 ± 0.6	1.0 ± 0.1	2.1 ± 0.1	3.7 ± 0.1
Navel	0	1.9 ± 0.1	5.5 ± 0.3	1.4 ± 0.0	2.1 ± 0.1	5.4 ± 0.2
**Control**						
5% DMSO	0			0		
Probineb 4.2 mg/mL	18.7 ± 0.2			20.5 ± 0.1		

Note: Data are shown as mean values of five replicates ± SD.

**Table 3:** MIC and MFC of orange oils against *C. gloeosporioides* and *C. scovillei*.

Essential Oils	MIC (% *v*/*v*)		MFC (% *v*/*v*)	
	*C. gloeosporioides*	*C. scovillei*	*C. gloeosporioides*	*C. scovillei*
Sanh	8	8	16	16
Xoan	4	4	16	16
Mat	4	4	16	16
Navel	8	8	16	16

**Table 4:** Disease inhibition of orange oils at various concentrations.

Essential Oil	Concentration (% *v*/*v*)	Disease Inhibition (%)					
		4 DAI		6 DAI		8 DAI	
		*C. g.*	*C. s.*	*C. g.*	*C. s.*	*C. g.*	*C. s.*
Xoan	4	11.1 ^d^	16.7 ^d^	24.3 ^e^	26.8 ^d^	22.4 ^e^	11.6 ^d^
Mat	4	10.4 ^d^	15.9 ^d^	29.3 ^d^	29.3 ^cd^	25.7 ^d^	12.8 ^d^
Navel	8	24.9 ^c^	25.3 ^c^	36.5 ^c^	32.1 ^c^	31.5 ^c^	21.3 ^c^
Sanh	8	24.3 ^c^	27.5 ^c^	38.3 ^c^	31.7 ^c^	31.4 ^c^	21.1 ^c^
Sanh	16	42.3 ^b^	45.3 ^b^	49.4 ^b^	47.5 ^b^	39.5 ^b^	30.4 ^b^
**Control**							
Distilled water		0	0	0	0	0	0
Probineb 4.2 mg/mL		59.7 ^a^	62.2 ^a^	58.1 ^a^	56.1 ^a^	52.1 ^a^	47.8 ^a^

Note: DAI: day after inoculation; *C. g.: Colletotrichum gloeosporioides*; *C. s.: Colletotrichum scovillei*. Different letters in the same column indicate significant differences at 5%.

**Table 5:** Quality characteristics of mangoes under the effect of Sanh orange oil.

EO Concentration (%)	Quality Parameters						
	pH	TSS (%)	TA (%)	Firmness (kgf/cm^2^)	Color		
					L^*^	a^*^	b^*^
**2**	4.67	14.1	0.72	2.56 ^a^	66.5	9.6 ^bc^	38.5 ^b^
**4**	4.94	14.8	0.73	2.55 ^a^	66.1	8.4 ^c^	41.9 ^ab^
**8**	5.01	14.5	0.73	2.42 ^a^	63.6	7.5 ^c^	44.3 ^a^
**16**	4.24	14.8	0.71	2.47 ^a^	63.0	8.4 ^c^	43.3 ^ab^
**Control**							
5% DMSO	4.82	14.1	0.72	2.15 ^b^	66.5	11.2 ^ab^	43.2 ^ab^
Distilled water	5.12	14.4	0.72	2.07 ^b^	66.7	12.8 ^a^	43.4 ^ab^

Note: Different letters in the same column indicate significant differences at 5%.

**Table 6:** Weight loss (%) in mangoes under the effect of Sanh orange oil.

EO Concentration (%)	Weight Loss (%)			
	2 DAT	4 DAT	6 DAT	8 DAT
**2**	3.32 ^c^	4.42 ^d^	5.95 ^d^	7.87 ^b^
**4**	3.65 ^c^	5.27 ^cd^	6.70 ^cd^	8.82 ^b^
**8**	6.58 ^a^	9.07 ^a^	10.98 ^a^	12.20 ^a^
**16**	6.14 ^a^	8.62 ^a^	10.54 ^a^	12.20 ^a^
**Control**				
DMSO 5%	4.60 ^b^	6.39 ^bc^	8.01 ^bc^	9.13 ^b^
Distilled water	4.74 ^b^	6.70 ^b^	8.49 ^b^	9.77 ^b^

Note: Different letters in the same column indicate significant differences at 5%. DAT: days after treatment.

## Data Availability

Not applicable.
